# A real-world pharmacovigilance study of KRAS G12C mutation inhibitors based on the food and drug administration adverse event reporting system

**DOI:** 10.3389/fphar.2024.1418469

**Published:** 2024-08-28

**Authors:** Lisha Wu, Maosheng Xu, Xueqin Li, Dilinuer Aierken, Jinxiu Yu, Tao Qin

**Affiliations:** ^1^ Department of Oncology, Sun Yat-sen Memorial Hospital, Sun Yat-sen University, Guangzhou, Guangdong, China; ^2^ Department of Oncology, Shenshan Medical Center, Sun Yat-sen Memorial Hospital, Sun Yat-sen University, Shanwei, Guangdong, China; ^3^ Department of Neurosurgery, Huashan Hospital, Fudan University, Shanghai, China

**Keywords:** adverse events, FAERS, disproportionality analysis, pharmacovigilance, sotorasib, adagrasib

## Abstract

**Introduction:**

Sotorasib and adagrasib have been widely used for the non-small cell lung cancer (NSCLC) patients harboring Kirsten rat sarcoma viral oncogene homolog (KRAS) G12C mutation. It's necessary to assess their safety profiles in the real-world population.

**Methods:**

A retrospective pharmacovigilance was conducted to examine adverse events (AEs) associated with sotorasib and adagrasib therapies using the US Food and Drug Administration (FDA) Adverse Event Reporting System (FAERS). Disproportionality analysis was performed employing Venn analysis and four data-mining algorithms, including the reporting odds ratio (ROR), the proportional reporting ratio (PRR), the Bayesian confidence propagation neural network (BCPNN), and the multi-item gamma Poisson shrinker (MGPS).

**Results:**

The most commonly reported system organ classes (SOCs) for both adagrasib and sororasib were general, gastrointestinal, and investigations disorders. Notably, sotorasib exhibited significant signals for neoplasms and hepatobiliary disorders in four algorithms. Specifically, AEs related to neoplasms were predominantly associated with lung malignancies, all of which were consistent with the therapeutic indications of KRAS G12C mutation inhibitor. A total of 19 common AEs were identified in sotorasib and adagrasib, spanning gastrointestinal, general, hepatobiliary, investigations, metabolism, musculoskeletal, neoplasms, and respiratory disorders. 4 severe AEs (SAEs) were identified in sotorasib, with 3 SAEs displaying significant signals in four algorithms, including drug-induced liver injury, pancreatitis, and hepatic failure. In adagrasib, only 2 SAEs were detected, with renal failure showing significant signals in four algorithms.

**Conclusion:**

This study offers a comprehensive evaluation of the major safety signals associated with sotorasib and adagrasib, providing valuable information for clinicians regarding drug selection and safety considerations, thereby facilitating the design of future prospective safety studies.

## 1 Introduction

The Kirsten rat sarcoma viral oncogene homolog (KRAS) encodes a membrane-bound guanosine triphosphatase (GTPase), which serves as a pivotal regulator in signal transduction cascades (Simanshu et al., 2017). GTPases, acting as molecular switches, catalyze the hydrolysis of GTP to GDP and regulate downstream activities by transitioning between a GTP-bound activated state and a GDP-bound inactive state (Vetter and Wittinghofer, 2001; Bos et al., 2007). KRAS mutations reduce the rate of GTP hydrolysis, leading to sustained activation of mutant proteins. This results in the continuous transmission of signals to downstream proteins, directing several different pathways in an uncontrolled manner, and showing a significant impact on tumorigenesis (Mustachio et al., 2021). The major KRAS mutations, including G12C, G12D, and G12V, are crucial drivers in the development of multiple tumor types (Liu et al., 2022). Particularly, the G12C stands out as the one of most common mutations in non-small cell lung cancer (NSCLC) patients, accounting for approximately 14% of non-squamous NSCLC (Lee et al., 2022a).

Sotorasib (AMG-510), a covalent inhibitor specific to KRAS G12C mutation, breaking the shackle that KRAS mutated patients have no target medicine for more than 30 years (Huang et al., 2021). The multicenter, single-arm, open-label Phase I/II trial (CodeBreak 100) demonstrated promising effects of sotorasib on locally advanced or metastatic KRAS G12C-mutated NSCLC patients previously received standard treatments. The results showed an objective response rate (ORR) of 37.1%, a median duration of response (DOR) of 11.1 months, a median progression-free survival (PFS) of 6.8 months, and a median overall survival (OS) of 12.5 months (Skoulidis et al., 2021). Sotorasib received FDA approval for the treatment of NSCLC patients with KRAS G12C mutations since May 2021, and was the world’s first targeted drug for KRAS mutations (Nakajima et al., 2022). Adagrasib (MRTX849), the second potent inhibitor of the KRAS G12C mutation, was also approved by the FDA in December 2022 for the treatment of KRAS G12C mutated locally advanced or metastatic NSCLC. The efficacy of adagrasib was observed in the Phase I/II KRYSTAL-1 trial, with an ORR of 42.9%, a median PFS of 6.5 months, and a median OS of 12.6 months (Jänne et al., 2022).

It is essential to investigate the safety profiles of KRAS G12C mutation inhibitors given their widespread application in NSCLC patients. However, due to limited follow-up time, selected populations, and lack of statistical power, clinical trials may not capture all aspects of sotorasib or adagrasib related adverse reactions in the real world. In particular, the Food and Drug Administration Adverse Event Reporting System (FAERS) serves as the largest publicly accessible pharmacovigilance databases for detection the adverse drug reactions (ADRs) of recently marketed drugs (FDA, 2018; Giunchi et al., 2023). Through its spontaneous reporting mechanism, FAERS is more efficient to identify AEs and provide accurate information among large population compared to clinical trials (Kumar, 2019). In this study, we conduct a real-world pharmacovigilance study to assess the AEs via FAERS data mining, for the purpose of providing comprehensive reference and theoretical guidance for the sotorasib and adagrasib safety in the clinical practice.

## 2 Method

### 2.1 Data source and data mining

We performed the retrospective study based on the FDA Adverse Event Reporting System (FAERS). The keywords (Sotorasib, Lumakras, AMG-510) were used for data mining. Data covering the period from April 2021 to December 2023 were cleaned and analyzed via SAS9.4 software. Similarly, the keywords (Adagrasib, Krazati, MRTX849) were used for AE cases related to adagrasib treatment, covering September 2022 to December 2023. Data were cleaned by de-duplication and excluding missing values according to the method recommended by the FDA. CaseID represented the patient’s identification, FDA_DT indicated the date of FDA report acceptance, and PRIMARYID denoted the unique report ID. A patient might submit multiple reports to the FDA at different time points. Reports with the same CASEID were sorted by CASEID, FDA_DT, and PRIMARYID. The report with the highest FDA_DT value among those with the same CASEID was retained. For reports with the same CASEID and FDA_DT, the one with the highest PRIMARYID value was retained. The de-duplicated data had unique CaseID and PRIMARYID values, ensuring accurate analysis (Khaleel et al., 2022). AE names in the FAERS database were described using the preferred terms (PT) from the Medical Dictionary for Regulatory Activities (MedDRA), which was updated each year (MedDRA, 2024). The updated system organ class (SOC) and preferred terms (PT) were obtained from the latest version of MedDRA for subsequent analysis. AEs related to “product issues”, “injury, poisoning and procedural complications”, “social circumstances” and “surgical and medical procedures” were not shown in the study for which were not drug related AEs (Fang et al., 2023).

### 2.2 Statistical analysis

This pharmacovigilance study conducted disproportionality analysis, which involved assessing the frequency of AEs associated with a specific drug compared to all other pharmaceutical agents (FDA, 2005). Disproportionality analysis was a critical analytical tool in pharmacovigilance research for identifying drug-related safety signals. In order to identify statistical associations between sotorasib and all AEs, the four major algorithms were used for data-mining (Zhang et al., 2023): the reporting odds ratio (ROR), the proportional reporting ratio (PRR), the Bayesian confidence propagation neural networks of information component (IC), and the empirical Bayes geometric mean (EBGM). The criteria for these four algorithms were shown in Table 1. Particularly, ROR was the key indicator for evaluating safety signals. We performed Venn analysis to differentiate common AEs from drug-specific ones. Microsoft EXCEL 365 and GraphPad Prism 8 software were employed for the major parts of statistical analysis.

**TABLE 1:** Four main algorithms used to calculate the safety signals of sotorasib and adagrasib.

Algorithms	Equation	Criteria
ROR	$\text{ROR}=\frac{a/c}{b/d}=\frac{\text{ad}}{\text{bc}}$	Lower limit of 95% CI > 1, N ≥ 3
	${\;95\%\text{CI}=e}^{\ln\left(\text{ROR}\right)\pm 1.96\sqrt{\left(\frac{1}{a}+\frac{1}{b}+\frac{1}{c}+\frac{1}{d}\right)}}$	
PRR	$\text{PRR}=\frac{a/\left(a+b\right)}{c/\left(c+d\right)}$	PRR ≥2, χ^2^ ≥ 4, N ≥ 3
	$\chi^{2}=\frac{{\left(\text{ad}-\text{bc}\right)}^{2}\left(a+b+c+d\right)}{\left(a+b\right)\left(a+c\right)\left(c+d\right)\left(b+d\right)}$	
IC	${\text{IC}=\log}_{2}\frac{a\left(a+b+c+d\right)}{\left(a+b\right)\left(a+c\right)}$	IC025 > 0
	$95\%\text{CI}=E\left(\text{IC}\right)\pm 2V\left(\text{IC}\right)0.5$	
MGPS	$\text{EBGM}=\frac{a\left(a+b+c+d\right)}{\left(a+c\right)\left(a+b\right)}$	EBGM05 > 2
	$95\%\text{CI}=e^{\ln\left(\text{EGBM}\right)\pm 1.96\left(\sqrt{\frac{1}{a}+\frac{1}{b}+\frac{1}{c}+\frac{1}{d}}\right)}$	

ROR, reporting odds ratio; PRR, proportional reporting ratio; IC, bayesian confidence propagation neural networks of information component; EBGM, empirical Bayes geometric mean; a, number of reports containing both the target drug and target adverse drug reaction; b, number of reports containing other adverse drug reaction of the target drug; c, number of reports containing the target adverse drug reaction of other drugs; d, number of reports containing other drugs and other adverse drug reactions; CI, confidence interval; N, number of reports; χ2, chi-squared; IC025, the lower limit of 95% CI, of the IC; E (IC), the IC, expectations; V(IC), the variance of IC; EBGM05, the lower limit of 95% CI, of EBGM.

## 3 Results

### 3.1 Population characteristics of sotorasib and adagrasib

Between April 2021 and December 2023, a total of 2028 cases, including 3588 AE reports following sotorasib administration, were obtained from the FAERS database. From September 2022 to December 2023, there were 338 cases, including 895 AE reports, involving adagrasib treatment. Table 2 presented the patient characteristics and AE reports for sotorasib and adagrasib. The proportions of female and male patients were nearly equal (35.75% vs. 34.17% and 28.99% vs. 25.15%, respectively). Patients aged 45 years and older constituted the majority of AE reports for sotorasib (42.85%). However, the age distribution of adagrasib was unclear due to over 90% missing data. From 2021 to 2023, the proportion of all AE reports showed an upward trend. The reporters of AEs for sotorasib and adagrasib were primarily physicians (55.23% vs. 24.26%), pharmacists (25.15% vs. 26.04%), and consumers (16.52% vs. 49.70%). The top three regions reporting adverse reactions were North America (37.73% vs. 83.14%), Europe (35.36% vs. 11.24%), and Asia (16.81% vs. 2.66%). The most frequent adverse reaction outcome for sotorasib was classified as “other serious” (52.61%), followed by “life-threatening or death” (24.56%) and “hospitalization” (18.49%). For adagrasib, the most common adverse outcomes were “life-threatening or death” (42.9%), “hospitalization” (41.12%), and “other serious” (16.27%). The analysis of time-to-onset revealed that most AEs occurred within the first 60 days of starting the drugs, whereas the missing data for time-to-onset exceeded 70%.

**TABLE 2:** Clinical characteristics of reports associated with sotorasib and adagrasib [n (%)].

	Sotorasib	Adagrasib
Sex		
Female	725 (35.75)	98 (28.99)
Male	693 (34.17)	85 (25.15)
Not Specified	610 (30.08)	155 (45.86)
Age		
<18	0 (0.00)	0 (0.00)
≥18, <45	16 (0.79)	1 (0.30)
≥45, <65	329 (16.22)	13 (3.85)
65≤	540 (26.63)	13 (3.85)
Not Specified	1,143 (56.36)	311 (92.01)
Year		
2021	146 (7.20)	/
2022	617 (30.42)	5 (1.48)
2023	1,265 (62.38)	333 (98.52)
Reporter		
Consumer	335 (16.52)	168 (49.70)
Not Specified	63 (3.11)	/
Pharmacist	510 (25.15)	88 (26.04)
Physician	1,120 (55.23)	82 (24.26)
Region		
North America	765 (37.72)	281 (83.14)
Europe	717 (35.36)	38 (11.24)
Asia	341 (16.81)	9 (2.66)
Oceania	36 (1.78)	1 (0.30)
South America	9 (0.44)	/
Not Specified	160 (7.89)	9 (2.66)
Outcome		
Life-Threatening/Death	498 (24.56)	5 (42.9)
Hospitalization	375 (18.49)	139 (41.12)
Disability	10 (0.49)	4 (1.18)
Required Intervention	2 (0.10)	0 (0.00)
Other serious	1,067 (52.61)	55 (16.27)
Time onset		
0-30d	174 (8.58)	112 (33.14)
31-60d	103 (5.08)	26 (7.69)
61-90d	63 (3.11)	8 (2.37)
91-120d	30 (1.48)	16 (4.73)
121-150d	17 (0.84)	4 (1.18)
151-180d	18 (0.89)	2 (0.59)
181-360d	40 (1.97)	5 (1.48)
>360d	21 (1.04)	0 (0.00)
missing value	1,562 (77.02)	165 (48.82)

### 3.2 SOC spectrum of sotorasib and adagrasib

**FIGURE 1:**
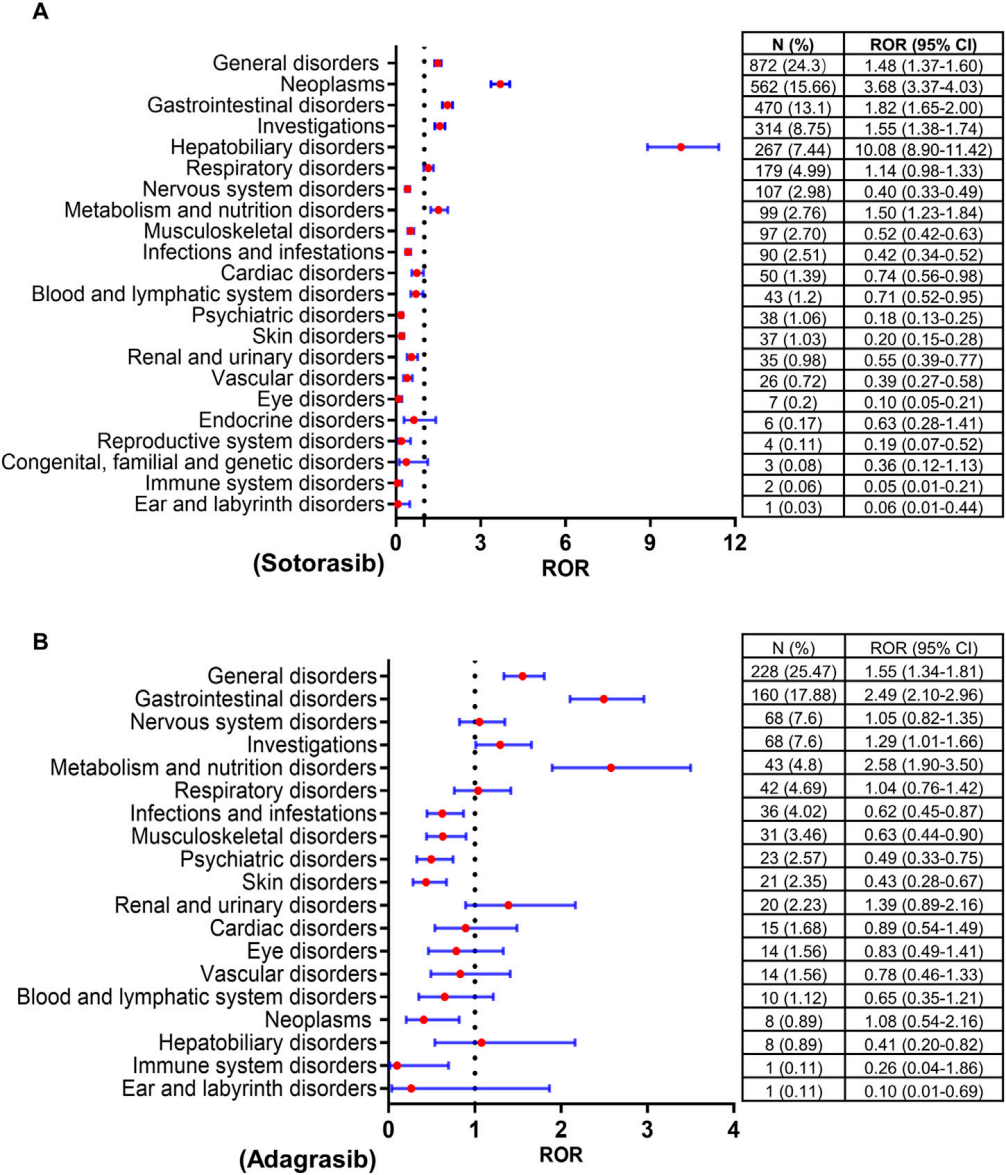
Forrest plots of the SOCs of sotorasib **(A)** and adagrasib **(B)**. SOC, system organ classification; ROR, reporting odds ratio; N, number of reports; CI, confidence interval..

Disproportionality analysis was conducted at the SOC level to identify safety signals for AEs associated with both drugs, as illustrated in Figure 1. The top five reported SOCs for sotorasib were general disorders (24.3%), neoplasms (15.66%), gastrointestinal disorders (13.1%), investigations (8.75%), and hepatobiliary disorders (7.44%). Similarly, the most frequently reported SOCs for adagrasib included general disorders (25.47%), gastrointestinal disorders (17.88%), nervous system disorders (7.6%), investigations (7.6%), and metabolism and nutrition disorders (4.8%). Both sotorasib and adagrasib exhibited safety signals in general, gastrointestinal, investigations and metabolism disorders (lower limit of ROR 95% CI > 1 and N ≥ 3). Particularly, sotorasib exhibited significant signals for neoplasms and hepatobiliary disorders in four algorithms (ROR, PRR, IC and EBGM).

### 3.3 PT spectrum of sotorasib and adagrasib

There were total 68 PTs across 13 SOCs exhibited safety signals corresponding to sotorasib-induced AEs upon the calculations of four algorithms. The PTs (report cases exceeding 15) were described in Supplementary Table S1. In our study, the most prevalent PTs associated with neoplasms were non-small cell lung cancer (304), non-small cell lung cancer metastatic (56), and lung neoplasm malignant (39). The top three reported PTs corresponding to hepatobiliary disorder were hepatotoxicity (55), hepatic function abnormal (44), and hepatic cytolysis (28). Similarly, the most reported PTs in investigation were aspartate aminotransferase increased (43), alanine aminotransferase increased (43), and liver function test increased (34). In term of gastrointestinal disorder, diarrhoea (196) and colitis (16) were notable. As for respiratory disorder, pulmonary embolism (19) and pneumonitis (17) represented the most common seen PTs. Regarding to general disorders, the top three reported PTs were disease progression (281), death (203), and adverse event (26).

Subsequent analysis indicated that adagrasib-related 22 PTs across 11 SOCs showed safety signals in four algorithms. The PTs (report cases exceeding 4) were described in Supplementary Table S2. The most prevalent PTs associated with general disorder were death (126), asthenia (25). The top three reported PTs related to gastrointestinal disorder were nausea (42), diarrhoea (41) and vomiting (30). Regarding to respiratory disorder, the most reported PTs were dyspnoea (16). Decreased appetite (15) and dehydration (11) exhibited signals in metabolism disorder. Dizziness (13) and seizure (9) were seen in nervous system disorders. The most reported PTs in investigation were weight decreased (9), blood creatinine increased (7), and electrocardiogram QT prolonged (6). Besides, renal failure (7) in renal and urinary disorders, hypotension (7) in vascular disorders, sepsis (6) in infections also showed signals, respectively.

### 3.4 Common AEs of sotorasib and adagrasib

Using Venn analysis, 19 common AEs with safety signals were identified in sotorasib and adagrasib across four algorithms. These AEs span multiple categories: gastrointestinal disorders (diarrhea, nausea, and vomiting); general disorders (death, general physical health deterioration, peripheral edema, and edema); hepatobiliary disorders (hepatotoxicity); investigations (increased aspartate aminotransferase, increased alanine aminotransferase, increased hepatic enzyme, increased gamma-glutamyltransferase, and increased blood creatinine); metabolism disorders (decreased appetite and dehydration); musculoskeletal disorders (myalgia); neoplasms (neoplasm progression); and respiratory disorders (pneumonitis and pleural effusion), as shown in Table 3.

**TABLE 3:** Common AEs between sotorasib and adagrasib.

SOC	PT	Sotorasib		Adagrasib	
		N	ROR	N	ROR
Gastrointestinal disorders	Diarrhoea	196	5.54	41	4.49
	Nausea	69	1.75	42	4.24
	Vomiting	33	1.47	30	5.36
General disorders	Death	203	4.32	126	12.05
	General physical health deterioration	14	2.10	7	4.12
	Oedema peripheral	9	2.09	6	5.47
	Oedema	8	3.33	3	4.62
Hepatobiliary disorders	Hepatotoxicity	55	39.18	3	9.02
Investigations	AST increased	43	20.52	5	8.87
	ALT increased	43	17.11	4	5.96
	Hepatic enzyme increased	34	8.67	4	3.94
	GGT increased	16	20.49	4	20.53
	Blood creatinine increased	7	2.14	7	8.50
Metabolism disorders	Decreased appetite	35	2.66	15	4.43
	Dehydration	14	2.38	11	7.35
Musculoskeletal disorders	Myalgia	18	2.37	5	2.43
Neoplasms	Neoplasm progression	11	3.57	6	7.52
Respiratory disorders	Pneumonitis	17	10.32	5	11.83
	Pleural effusion	15	5.45	3	4.47

AE, adverse event; SOC, system organ classification; PT, preferred terms; N, number of reports; ROR, reporting odds ratio; AST, aspartate aminotransferase; ALT, alanine aminotransferase; GGT, gamma-glutamyltransferase.

### 3.5 Disproportionality analysis of SAEs

Total 4 SAEs were identified in FAERS database related to sotorasib treatment, shown in Table 4, with 3 SAEs exhibiting significant signals in the four algorithms and reported number larger than three. These 3 SAEs associated with sotorasib treatment were drug-induced liver injury [ROR (95% CI) = 5.92 (3.36–10.44), n = 12], pancreatitis [ROR (95% CI) = 5.10 (2.74–9.50), n = 10] and hepatic failure [ROR (95% CI) = 7.61 (3.95–14.64), n = 9]. There were only 2 SAEs detected in adagrasib, and renal failure [ROR (95% CI) = 5.18 (2.46–10.91), n = 7] exhibited significant signals in four algorithms, shown in Table 5.

**TABLE 4:** SAEs cases for sotorasib exhibited safety signals in four algorithms.

SAE	N	ROR (95% CI)	PRR (x2)	IC (IC025)	EGBM (EGBM05)
Drug-induced liver injury	12	5.92 (3.36–10.44)	5.90 (48.83)	2.56 (1.30)	5.90 (3.34)
Pancreatitis	10	5.10 (2.74–9.50)	5.09 (32.86)	2.35 (1.02)	5.09 (2.73)
Hepatic failure	9	7.61 (3.95–14.64)	7.59 (51.42)	2.92 (1.28)	7.58 (3.84)
Acute hepatic failure	3	4.19 (1.35–13.01)	4.19 (7.27)	2.07 (−0.22)	4.18 (1.35)

SAE, severe adverse event; N, number of reports; ROR, reporting odds ratio; PRR, proportional reporting ratio; IC, bayesian confidence propagation neural networks of information component; EBGM, empirical Bayes geometric mean; CI, confidence interval; N, number of reports; χ2, chi-squared; IC025, the lower limit of 95% CI, of the IC; EBGM05, the lower limit of 95% CI, of EBGM.

**TABLE 5:** SAEs cases for adagrasib exhibited safety signals in four algorithms.

SAE	N	ROR (95% CI)	PRR (χ2)	IC (IC025)	EBGM (EBGM05)
Renal failure	7	5.18 (2.46–10.91)	5.15 (23.43)	2.36 (0.74)	5.15 (2.45)
Acute kidney injury	7	2.95 (1.40–6.20)	2.93 (8.94)	1.55 (0.21)	2.93 (1.39)

SAE, severe adverse event; N, number of reports; ROR, reporting odds ratio; PRR, proportional reporting ratio; IC, bayesian confidence propagation neural networks of information component; EBGM, empirical Bayes geometric mean; CI, confidence interval; N, number of reports; χ2, chi-squared; IC025, the lower limit of 95% CI, of the IC; EBGM05, the lower limit of 95% CI, of EBGM.

## 4 Discussion

KRAS mutation is a pivotal driver gene of NSCLC. Its complex spatial structure has historically rendered KRAS mutation the most challenging target for drug development (Huang et al., 2021). Sotorasib and adagrasib have emerged as promising agents for precision-targeted therapy in KRAS-mutant NSCLC, with several clinical trials underway for drugs targeting KRAS G12C mutation (Skoulidis et al., 2021; Jänne et al., 2022; Hong et al., 2020; de Langen et al., 2023). Our study represents the inaugural long-term pharmacovigilance investigation utilizing real-world data to assess the safety profiles of sotorasib and adagrasib. Our study provides a comprehensive overview of the major safety signals associated with KRAS G12C mutantation inhibitors, offering valuable insights for clinicians in drug selection and safety considerations, paving the way for future prospective safety studies.

Based on clinical research data and retrospective analysis, hepatotoxicity is a significant adverse reaction associated with KRAS G12C mutation inhibitors treatment. In the CodeBreak200 study, sotorasib-induced liver adverse reactions manifested as elevated levels of alanine aminotransferase (ALT) (10% for any grade, 8% for grade 3 or higher) and aspartate aminotransferase (AST) (10% for any grade, 5% for grade 3 or higher) (de Langen et al., 2023). In the KRYSTAL-1 study, the increased levels of ALT (28.4% for any grade, 5.2% for grade 3 or higher) and AST (26.7% for any grade, 5.2% for grade 3 or higher) also represented the one of the most common AEs (Jänne et al., 2022). These findings align with results obtained from our analysis of real-world data. The precise underlying mechanism of KRAS G12C mutation inhibitors induced liver injury is currently unclear. Post hoc analysis of CodeBreaK200 revealed a higher incidence of severe liver adverse events in patients treated with sotorasib 1–2.6 months after receiving treatment with immune checkpoint inhibitors. Studies revealed the correlation between prior use of programmed death-1 (PD-1)/programmed death-ligand 1 (PD-L1) and severe hepatotoxicity associated with sotorasib. The short intervals, history of previous immune-related hepatitis, and high plasma concentrations of anti-PD-1 were identified as key factors (Ernst et al., 2024; Chour et al., 2023). Preclinical investigation suggested that sotorasib might induce an inflammatory tumor microenvironment, increasing infiltration of CD8^+^ T cells (Canon et al., 2019), which could be a contributing factor to the occurrence of immune-related hepatotoxicity following combination or sequential use of sotorasib with immune checkpoint inhibitors. The use of corticosteroids agents should be considered in addition to discontinuing treatment in cases of severe hepatotoxicity (Garassino et al., 2023). Additionally, reports suggest the feasibility and safety of sequential adagrasib treatment in patients who encountered grade 3 sotorasib-related hepatotoxicity and discontinued sotorasib (Luo et al., 2024). Adagrasib’s distinct off-target effects and pharmacokinetic profiles compared to sotorasib supported this transition (Ou et al., 2022; Wang et al., 2023).

Another significant adverse reaction of sotorasib and adagrasib is gastrointestinal disturbance. In our study, the most common symptoms of treatment-induced gastrointestinal disturbance were diarrhea, nausea, and vomiting. These findings were consistent with the CodeBreak100 clinical study, where the rates of diarrhea, nausea, and vomiting were 29.5%, 20.9%, and 17.8%, respectively (Hong et al., 2020). Similarly, the Codebreak200 study reported diarrhea, nausea, and vomiting at rates of 34%, 24%, and 5%, respectively (de Langen et al., 2023). The rates were much higher in the KRYSTAL-1 study, where diarrhea, nausea, and vomiting represented 70.7%, 69.8% and 56.9%, respectively (Jänne et al., 2022). The precise mechanism of KRAS G12C mutation inhibitor-related gastrointestinal toxicity is still unknown. A multicenter retrospective study examining the clinical characteristics of advanced non-small cell lung cancer patients with KRAS G12C mutation found an association between sotorasib toxicity and recent exposure to immune checkpoint inhibitors, which could also explain the occurrence of sotorasib-induced enteritis (Thummalapalli et al., 2023).

Our study findings indicated that another primary adverse reaction associated with KRAS G12C mutation inhibitors were respiratory system related. Specifically, AEs related to tumors associated with sotorasib were predominantly linked to lung malignancies, including non-small cell lung cancer (NSCLC), NSCLC metastatic, malignant lung neoplasms, and lung adenocarcinomas, all of which align with the therapeutic indications of sotorasib. Our results revealed that pneumonitis and pleural effusion were the common AEs in respiratory disorders. Fatal events of respiratory system were rare. In the Codebreak200, only one case (<1%) in the sotorasib cohort reported fatal interstitial lung disease. One case in the KRYSTAL-1 reported fatal pulmonary hemorrhage. Beyond the established correlation between exposure to anti-PD-(L)1 therapy and KRAS G12C mutation inhibitor related-toxicity, parallels may also exist with severe adverse events observed with tyrosine kinase inhibitors (Shah, 2016). Given the role of KRAS as a downstream factor in the receptor tyrosine kinases (RTKs) pathway, which contributes to the activation of receptor pathways such as epidermal growth factor receptor (EGFR) and human epidermal growth factor receptor-2 (Her-2) (Heppner and Eck, 2021; Zenonos and Kyprianou, 2013), and considering the involvement of these membrane surface receptors in the growth and repair of airway epithelial cells as well as lung injury repair, it is plausible to infer an association between KRAS inhibitors and interstitial pneumonia. Notably, the predominant demographic among individuals with KRAS-mutant NSCLC comprises males with a history of smoking (Wang et al., 2021; Lee et al., 2022b), of which recognized as high-risk factor for interstitial pneumonia (Dawod et al., 2020; Choi et al., 2018).

There are several limitations to our study. Firstly, the number of adverse reactions to specific drugs may be influenced by factors such as drug dosage, reporting populations, and the nature of adverse reactions, which could impede the accurate reflection of all adverse reactions caused by sotorasib and adagrasib in our study. Secondly, the FAERS database may not capture complete information, resulting in numerous clinical data gaps including patient status, comorbidities, and treatment indications, thereby compromising result accuracy and introducing bias. Thirdly, the lack of comprehensive data from all individuals makes it impossible to determine the incidence of adverse reactions. Fourthly, disproportionality analysis can only evaluate signal strength, thus incapable of quantifying risk or establishing causality. Lastly, our study suffers from a limited number of reports on sotorasib and adagrasib, necessitating additional reports or larger-scale clinical studies for further validation.

## 5 Conclusion

In conclusion, we conducted the disproportionality analysis of KRAS G12C mutation inhibitors induced AEs according to the FAERS database. Data were collected between April 2021 and December 2023. The three most frequently reported SOCs of adagrasib and sororasib were general, gastrointestinal, and investigations disorders. Sotorasib showed significant signals for neoplasms and hepatobiliary disorders in four algorithms. Specifically, AEs related to tumors are predominantly linked to lung malignancies, all of which align with the therapeutic indications of sotorasib. There were 19 common AEs detected in sotorasib and adagrasib. Total 4 SAEs identified in sotorasib and 2 SAEs were detected in adagrasib, respectively. This comprehensive post-marketing safety surveillance significantly enhances the understanding of safety profiles of KRAS G12C mutation inhibitors, thereby offering valuable insights for studies and clinical practice in the future.

## Data Availability

The data is openly available in the FDA Adverse Event Reporting System Public Dashboard at: https://fis.fda.gov/extensions/FPD-QDE-FAERS/FPD-QDE-FAERS.html. Further inquiries can be directed to the corresponding author.
