# Supplementary material for: A real-world pharmacovigilance study of KRAS G12C mutation inhibitors based on the food and drug administration adverse event reporting system
Source: Front Pharmacol. 2024 Aug 28;15:1418469. doi: 10.3389/fphar.2024.1418469 (PMC11387170; doi:10.3389/fphar.2024.1418469)
Supplement: Supplementary file 1 [file Table1.docx]

**Supplementary Table 1** Reports of sotorasib at the PT level exhibited safety signals in four algorithm and N≥15.

| **SOC** | **PT** | **N** | **ROR (95% CI)** | **PRR (χ2)** | **IC (IC025)** | **EGBM (EGBM05)** |
| --- | --- | --- | --- | --- | --- | --- |
| Neoplasms benign, malignant and unspecified | Non-small cell lung cancer | 304 | 974.73  (855.42-1109.87) | 891.90 (216627.90) | 9.48  (7.55) | 714.31 (627.10) |
|  | Non-small cell lung cancer metastatic | 56 | 1698.65  (1234.51-2336.11) | 1671.73 (63754.31) | 10.15 (5.32) | 1140.14 (828.81) |
|  | Lung neoplasm malignant | 39 | 8..3 (6.23-11.72) | 8.46 (256.28) | 3.08 (2.37) | 8.44 (6.16) |
|  | Lung adenocarcinoma | 23 | 145.42 (96.10-221.78) | 145.06 (3162.57) | 7.12 (3.76) | 139.45 (91.79) |
| Hepatobiliary disorders | Hepatotoxicity | 55 | 39.18 (29.98-51.21) | 38.59 (1993.46) | 5.26 (4.13) | 38.19 (29.22) |
|  | Hepatic function abnormal | 44 | 21.48 (15.94-28.95) | 21.23 (843.74) | 4.40 (3.43) | 21.11 (15.67) |
|  | Hepatic cytolysis | 28 | 18.06 (12.44-26.22) | 17.93 (445.50) | 4.16 (2.96) | 17.84 (12.29) |
|  | Liver disorder | 23 | 9.97 (6.61-15.03) | 9.91 (183.92) | 3.31 (2.26) | 9.89 (6.56) |
|  | Cholestasis | 20 | 22.20 (14.28-34.49) | 22.08 (400.12) | 4.46 (2.82) | 21.95 (14.12) |
| Gastrointestinal disorders | Diarrhoea | 196 | 5.54 (4.80-6.40) | 5.30 (689.25) | 2.40 (2.16) | 5.29 (4.58) |
|  | Colitis | 16 | 7.11 (4.35-11.63) | 7.09 (83.53) | 2.82 (1.68) | 7.07 (4.33) |
| Respiratory, thoracic and mediastinal disorders | Pulmonary embolism | 19 | 5.34 (3.40-8.38) | 5.31 (66.52) | 2.41 (1.48) | 5.31 (3.38) |
|  | Pneumonitis | 17 | 10.32 (6.40-16.63) | 10.27 (141.96) | 3.36 (2.08) | 10.25 (6.36) |
| Investigations | Aspartate aminotransferase increased | 43 | 20.52 (15.18-27.75) | 20.29 (784.59) | 4.33 (3.37) | 20.18 (14.93) |
|  | Alanine aminotransferase increased | 43 | 17.11 (12.65-23.12) | 16.91 (641.21) | 4.07 (3.19) | 16.84 (12.46) |
|  | Liver function test increased | 34 | 25.01 (17.82-35.10) | 24.78 (770.89) | 4.62 (3.39) | 24.62 (17.54) |
|  | Hepatic enzyme increased | 34 | 8.67 (6.18-12.16) | 8.60 (228.07) | 3.10 (2.33) | 8.58 (6.12) |
|  | Blood alkaline phosphatase increased | 18 | 22.84 (14.35-36.34) | 22.73 (371.63) | 4.50 (2.74) | 22.59 (14.20) |
|  | Gamma-glutamyltransferase increased | 16 | 20.49 (12.52-33.53) | 20.40 (293.65) | 4.34 (2.54) | 20.29 (12.40) |
| General disorders and administration site conditions | Disease progression | 281 | 37.03 (32.77-41.85) | 34.21 (8994.56) | 5.08 (4.74) | 33.90 (29.99) |
|  | Death | 203 | 4.32 (3.75-4.98) | 4.13 (488.49) | 2.05 (1.82) | 4.13 (3.58) |
|  | Adverse event | 26 | 6.61 (4.49-9.73) | 6.57 (122.73) | 2.71 (1.89) | 6.56 (4.46) |

SOC, system organ classification; N, number of reports; PT, preferred terms; ROR, reporting odds ratio; PRR, proportional reporting ratio; IC, Bayesian confidence propagation neural networks of information component; EBGM, empirical Bayes geometric mean; CI, confidence interval; N, number of reports; χ2, chi-squared; IC025, the lower limit of 95% CI of the IC; EBGM05, the lower limit of 95% CI of EBGM.

**Supplementary Table 2** Reports of adagrasib at the PT level exhibited safety signals in four algorithm and N≥5.

| **SOC** | **PT** | **N** | **ROR （95% CI）** | **PRR (χ2)** | **IC (IC025)** | **EBGM (EBGM05)** |
| --- | --- | --- | --- | --- | --- | --- |
| General disorders and administration site conditions | Death | 126 | 12.05 (9.98-14.56) | 10.50 (1095.89) | 3.39 (3.01) | 10.48 (8.86) |
|  | Asthenia | 25 | 5.07 (3.40-7.54) | 4.95 (79.29) | 2.31 (1.53) | 4.95 (3.0 |
|  | General physical health deterioration | 7 | 4.12 (1.96-8.67) | 4.10 (16.40) | 2.03 (0.54) | 4.09 (1.6 |
|  | Oedema peripheral | 6 | 5.47 (2.45-12.20) | 5.44 (21.73) | 2.44 (0.64) | 5.43 (2.5) |
| Gastrointestinal disorders | Nausea | 42 | 4.24 (3.11-5.78) | 4.09 (98.95) | 2.03 (1.48) | 4.08 (3.1 |
|  | Diarrhoea | 41 | 4.49 (3.28-6.14) | 4.33 (106.04) | 2.11 (1.55) | 4.33 (3.8 |
|  | Vomiting | 30 | 5.36 (3.72-7.71) | 5.21 (102.62) | 2.38 (1.67) | 5.21 (3.2 |
| Respiratory, thoracic and mediastinal disorders | Dyspnoea | 16 | 2.26 (1.38-3.71) | 2.24 (11.08) | 1.16 (0.35) | 2.24 (1.8 |
|  | Pneumonitis | 5 | 11.83 (4.91-28.52) | 11.77 (49.23) | 3.56 (0.89) | 11.76 (4.12) |
| Metabolism and nutrition disorders | Decreased appetite | 15 | 4.43 (2.66-7.38) | 4.37 (39.16) | 2.13 (1.12) | 4.37 (2.6 |
|  | Dehydration | 11 | 7.35 (4.05-13.32) | 7.27 (59.50) | 2.86 (1.41) | 7.26 (4.5) |
| Nervous system disorders | Dizziness | 13 | 2.05 (1.19-3.55) | 2.04 (6.91) | 1.03 (0.14) | 2.04 (1.9 |
|  | Seizure | 9 | 5.02 (2.60-9.68) | 4.98 (28.67) | 2.32 (0.91) | 4.98 (2.0 |
| Investigations | Weight decreased | 9 | 2.13 (1.10-4.11) | 2.12 (5.34) | 1.08 (0.01) | 2.12 (1.0 |
|  | Blood creatinine increased | 7 | 8.50 (4.04-17.89) | 8.44 (45.91) | 3.08 (1.10) | 8.43 (4.4) |
|  | Electrocardiogram QT prolonged | 6 | 13.10 (5.87-29.27) | 13.02 (66.51) | 3.70 (1.16) | 13.00 (5.77) |
|  | Aspartate aminotransferase increased | 5 | 8.87 (3.68-21.38) | 8.83 (34.69) | 3.14 (0.75) | 8.82 (3.8) |
| Renal and urinary disorders | Renal failure | 7 | 5.18 (2.46-10.91) | 5.15 (23.43) | 2.36 (0.74) | 5.15 (2.6) |
|  | Acute kidney injury | 7 | 2.95 (1.40-6.20) | 2.93 (8.94) | 1.55 (0.21) | 2.93 (1.0 |
| Vascular disorders | Hypotension | 7 | 2.72 (1.29-5.73) | 2.71 (7.58) | 1.44 (0.13) | 2.71 (1.9 |
| Neoplasms benign, malignant and unspecified | Neoplasm progression | 6 | 7.52 (3.37-16.80) | 7.48 (33.68) | 2.90 (0.86) | 7.47 (3.7) |
| Infections | Sepsis | 6 | 4.42 (1.98-9.87) | 4.40 (15.76) | 2.14 (0.47) | 4.40 (1.8 |
| Psychiatric disorders | Confusional state | 6 | 3.38 (1.51-7.54) | 3.36 (9.96) | 1.75 (0.23) | 3.36 (1.1 |

SOC, system organ classification; N, number of reports; PT, preferred terms; ROR, reporting odds ratio; PRR, proportional reporting ratio; IC, Bayesian confidence propagation neural networks of information component; EBGM, empirical Bayes geometric mean; CI, confidence interval; N, number of reports; χ2, chi-squared; IC025, the lower limit of 95% CI of the IC; EBGM05, the lower limit of 95% CI of EBGM.
